# An intimate view of a spliceosome component

**DOI:** 10.7554/eLife.06200

**Published:** 2015-02-13

**Authors:** Timothy W Nilsen

**Affiliations:** Center for RNA Molecular Biology, Case Western Reserve University, Cleveland, United Statestwn@case.edu

**Keywords:** pre-mRNA splicing, crystallography, spliceosome, U1 snRNP, 5′ splice site, human

## Abstract

A high-resolution structure reveals how the ribonucleoprotein particle called U1 snRNP engages with 5′ splice sites.

**Related research article** Kondo Y,Oubridge C, van Roon AM, Nagai K. 2015. Crystal structure of human U1 snRNP, a small nuclear ribonucleoprotein particle, reveals the mechanism of 5′ splice site recognition. *eLife* **4**:e04986. doi: 10.7554/eLife.04986

**Image** The U1 snRNP comprises a 164-base long RNA molecule bound to 10 proteins

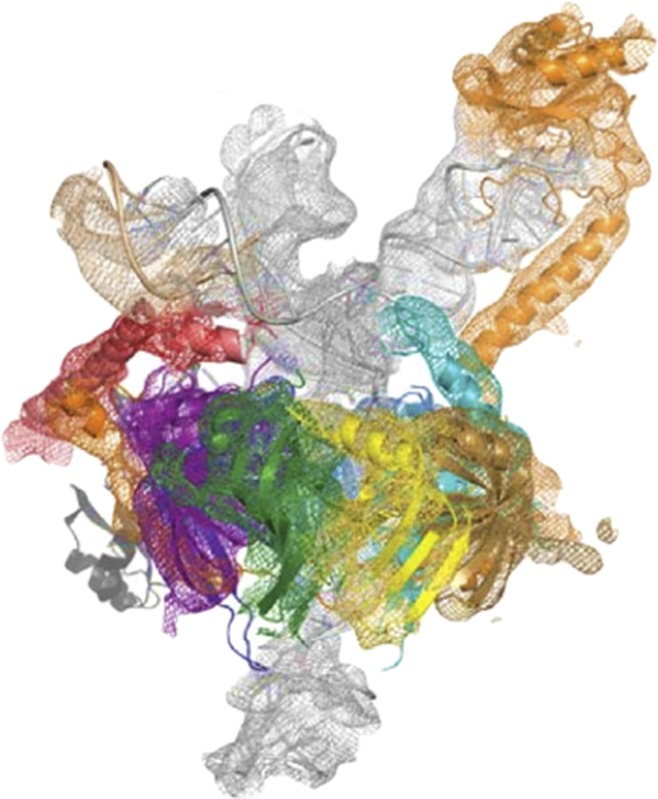

Gene expression is a carefully regulated process. Within the nucleus of a eukaryotic cell, multiple molecules work in concert to control whether or not a gene is transcribed to produce a molecule of pre-messenger RNA. Other molecules then direct how this molecule is processed to form a messenger RNA, and still more molecules determine if the messenger RNA is, in turn, translated to form a protein.

Molecular complexes containing short RNA molecules and proteins also play several essential roles in regulating gene expression in eukaryotes. The RNA molecules in these so-called small nuclear ribonucleoprotein particles (or snRNPs) are rich in uridine bases, so they are often known as ‘U snRNPs’. Five of these particles are required for a process called pre-messenger RNA splicing: this involves the removal of introns—regions of RNA that do not code for proteins—from the pre-messenger RNA. Because the vast majority of genes in higher eukaryotes contain introns, this process must take place for almost all messenger RNAs.

Splicing is carried out by a large ribonucleoprotein complex known as the spliceosome. Unlike ribosomes, the particles that translate messenger RNA, spliceosomes are not pre-formed but are assembled anew on each intron. Thirty-five years ago it was proposed that U1 snRNP recognizes the start, or 5′ end, of an intron (Lerner et al., 1980; Rogers and Wall, 1980); this was confirmed by experiments six years later (Zhuang and Weiner, 1986). The recognition of the 5′ splice site by U1 snRNP is now known to be the molecular event that initiates the assembly of the spliceosome.

Now, in *eLife*, Kiyoshi Nagai and colleagues at the MRC Laboratory of Molecular Biology—including Yasushi Kondo and Chris Oubridge as joint first authors—report a high-resolution crystallographic analysis of the structure of U1 snRNP. In doing so, they finally reveal in detail how this ribonucleoprotein particle engages 5′ splice sites (Kondo et al., 2015).

The U1 snRNP is comprised of one short RNA molecule, a proteinaceous ring (called the Sm ring) made of seven Sm proteins, and three more U1 snRNP-specific proteins (named U1-70k, U1-A, and U1-C). When viewed in two dimensions, most of the RNA molecule resembles a cloverleaf (because it folds back on itself to form three loops). The first loop is the binding site for the U1-70k protein, the second is the binding site for the U1-A protein, and the third makes extensive contacts with the Sm ring. The U1 RNA molecule also contains a fourth stem loop, but this is far removed from the business end of the snRNP.

Nagai and colleagues had previously determined the structure of U1 snRNP using X-ray crystallography to a moderate resolution of 5.5 Å (Pomeranz Krummel et al., 2009). Attempts to obtain a more detailed picture of the structure were unsuccessful, largely because the complex was too flexible to form the highly ordered crystals needed for higher resolution. However, guided by the existing structure, Nagai, Kondo, Oubridge and colleagues were able to cleverly split the snRNP into two smaller substructures, each of which produced more ordered crystals that diffracted to high resolution. One substructure contained the Sm ring, the U1-C protein, a fragment of the U1-70K protein, and a shortened version of the U1 RNA molecule. The fragment of U1-70K was included because it was known to make multiple protein–protein interactions with the Sm ring and the U1-C protein (which also makes many contacts with the Sm ring).

As expected a section near the beginning of the U1 RNA molecule bound, via base pairing, to a complementary sequence in a short RNA molecule that had been designed to mimic a 5′ splice site. Of more interest were contacts made between this double-stranded RNA structure (or duplex) and the U1-C protein. It had previously been reported that U1 snRNP lacking its starting sequence, and thus unable to base pair with the 5′ splice site, still selected 5′ splice site sequences from a pool of RNA molecules of random sequence (Du and Rosbash, 2002). This result was interpreted to mean that the U1-C was a sequence specific RNA-binding protein.

Nevertheless, in the high-resolution crystal structure, U1-C does not make any contacts with the bases of either the U1 RNA molecule or the 5′ splice site RNA. Instead, it makes multiple contacts with the sugar phosphate backbones of both RNA strands (Figure 1). These observations simultaneously rule out the notion that the U1-C protein is a sequence-specific RNA-binding protein and reveal the true role of U1-C in splice site recognition. That is to say that, via backbone interactions, U1-C stabilizes the duplex between the U1 RNA molecule and 5′ splice sties. This stabilization function explains why many 5′ splice site sequences, some of which are not completely complementary to the sequence in the U1 RNA, can still interact in a functionally significant way with the U1 snRNP.

**Figure 1.**
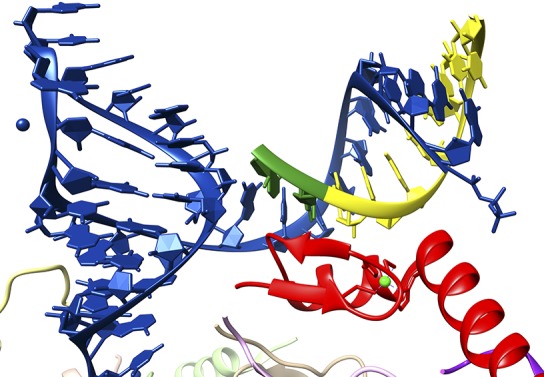
Close-up of the interaction between a 5′ splice site and the U1 snRNP. The crystal structure solved by Kondo, Oubridge et al. provides a detailed view of the interaction between the U1 RNA molecule (blue), an oligoribonucleotide that mimics a 5′ splice site (the intron is in yellow; the exon, which codes for protein, is in green) and the U1-C protein (red). It can be seen that U1-C does not make base specific contacts with either strand of the duplex formed by the U1 RNA and the splice site.

FIGURE CREDIT: IMAGE COURTESY OF KIYOSHI NAGAI.

In closing, recent studies have revealed three other functions for the U1 snRNP beyond splice site recognition. First, it prevents the poly(A) tail—which marks the end of a mature messenger RNA—from being added too early or at the wrong sites in a new pre-messenger RNA (Kaida et al., 2010). This activity likely results from the U1-70K protein antagonizing the enzyme that builds the poly(A) tail onto the messenger RNA (Gunderson et al., 1998). The second function, which may well be related to the first, is that U1 snRNP regulates precisely where a poly(A) tail is added to pre-messenger RNAs with more than one useable site (Berg et al., 2012). As such, this latter function determines how long the mature messenger RNA will be. Third, it is now known that most promoters in higher cells initiate RNA synthesis in both directions. U1 snRNP has a central role in ensuring that only RNA synthesis in the right direction is productive (Almada et al., 2013). As the mechanisms behind these activities are analyzed, the crystal structure of U1 snRNP will undoubtedly aid in the design and interpretation of future biochemical experiments.
